# Evidence for rapid downward fecundity selection in an ectoparasite (*Philornis downsi*) with earlier host mortality in Darwin’s finches

**DOI:** 10.1111/jeb.13588

**Published:** 2020-02-07

**Authors:** Lauren K. Common, Jody A. O’Connor, Rachael Y. Dudaniec, Katharina J. Peters, Sonia Kleindorfer

**Affiliations:** ^1^ College of Science and Engineering Flinders University Adelaide SA Australia; ^2^ Department for Environment and Water Government of South Australia Adelaide SA Australia; ^3^ Department of Biological Sciences Macquarie University Sydney NSW Australia; ^4^ Konrad Lorenz Research Center for Behaviour and Cognition and Department of Behavioural and Cognitive Biology University of Vienna Vienna Austria

**Keywords:** abdomen size, body size, Darwin's finches, Diptera, Galápagos Islands, host–parasite

## Abstract

Fecundity selection is a critical component of fitness and a major driver of adaptive evolution. Trade‐offs between parasite mortality and host resources are likely to impose a selection pressure on parasite fecundity, but this is little studied in natural systems. The ‘fecundity advantage hypothesis’ predicts female‐biased sexual size dimorphism whereby larger females produce more offspring. Parasitic insects are useful for exploring the interplay between host resource availability and parasite fecundity, because female body size is a reliable proxy for fecundity in insects. Here we explore temporal changes in body size in the myiasis‐causing parasite *Philornis downsi* (Diptera: Muscidae) on the Galápagos Islands under conditions of earlier in‐nest host mortality. We aim to investigate the effects of decreasing host resources on parasite body size and fecundity. Across a 12‐year period, we observed a mean of *c*. 17% *P. downsi* mortality in host nests with 55 ± 6.2% host mortality and a trend of *c*. 66% higher host mortality throughout the study period. Using specimens from 116 Darwin's finch nests (Passeriformes: Thraupidae) and 114 traps, we found that over time, *P. downsi* pupae mass decreased by *c*. 32%, and male (*c*. 6%) and female adult size (*c*. 11%) decreased. Notably, females had *c*. 26% smaller abdomens in later years, and female abdomen size was correlated with number of eggs. Our findings imply natural selection for faster *P. downsi* pupation and consequently smaller body size and lower parasite fecundity in this newly evolving host–parasite system.

## Graphical abstract

Frontal view of a female *Philornis downsi*, a myasis‐causing paraiste of Darwin's finches, whose body size, abdomen size, and fecundity have decreased *c*. 30% across 12 years as nestling hosts die at an ever younger age. Photo by Bradley Sinclair.

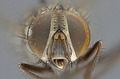

## 1 INTRODUCTION

Fecundity selection affects fitness by favouring traits associated with increased reproductive output (Roff, 2001). Few studies examine fecundity selection (Pincheira‐Donoso & Hunt, 2017) and those that do generally focus on traits that increase fecundity (upward selection) (Orozco & Bell, 1974; Preziosi & Fairbairn, 1996; Saino et al., 2017; Välimäki & Kaitala, 2007). Although traits that increase or decrease fecundity covary, far fewer studies have observed downward selection on traits leading to decreased fecundity (Nunney, 1996; Orozco & Bell, 1974; Quintero‐Fong et al., 2018; Reeve & Fairbairn, 1999). To better understand the role of fecundity selection on variation in biological fitness, we need case studies that identify temporal patterns and processes of fecundity change. Host–parasite systems make excellent candidates for such case studies given their tight co‐evolutionary interactions that depend on fecundity and survival. Thus, the relationship between parasite virulence and host mortality can be explored to understand the drivers and direction of fecundity selection.

The ‘fecundity advantage hypothesis’ was originally formulated by Darwin (1871) to explain the common occurrence of large female body size (Cox, Skelly, & John‐Alder, 2003; Shine, 1989). Across taxa, female body size is positively associated with fecundity (Pincheira‐Donoso & Hunt, 2017), as larger‐bodied females can physically accommodate more offspring and can store more energy to invest in reproduction (Calder, 1996). Strong positive fecundity selection can generate directional selection for increased female body size in insects (Andersen, 1994; Hurlbutt, 2008; Sivinski & Dodson, 1992; Teder & Tammaru, 2005) and other taxa (Braña, 1996; Scharf & Meiri, 2013), and can also result in the increased size of particular body regions (i.e. trunk or abdomen) that are functionally linked to fecundity (Olsson, Shine, Wapstra, Ujvari, & Madsen, 2002; Parker et al., 2011; Preziosi, Fairbairn, Roff, & Brennan, 1996; Winkler, Stölting, & Wilson, 2012). Parasitic insects provide useful systems to test ideas about effects of body size on fecundity because parasite diets can be tracked through host availability (Nijhout, 2003; Lahuatte, Lincango, Heimpel, & Causton, 2016). In this way, parasitic insects can provide insights into changing body size and fecundity with altered nutritional conditions.

Parasites must balance virulence and fitness with maximizing host resource use to ensure life cycle completion before host death (Hatcher, Dick, & Dunn, 2012). Increased host exploitation may lead to larger body size and higher fecundity, but could result in early termination of the host and eventually population collapse as host populations are exhausted (Hatcher et al., 2012). Recent host–parasite associations undergoing co‐evolutionary interactions are therefore ideal case studies for examining changing fecundity selection under unstable host resource pressures.

Here we focus on natural selection for small body size in the fly, *Philornis downsi* (Diptera: Muscidae) (Dodge and Aitken), which is an invasive myiasis‐causing parasite of Darwin's finches on the Galápagos Islands. *Philornis downsi* larvae consume the blood and tissue of nestling birds, causing up to 100% in‐nest mortality in some of its Darwin's finch hosts (Dudaniec & Kleindorfer, 2006; Fessl, Heimpel, & Causton, 2018; Kleindorfer, Peters, Custance, Dudaniec, & O’Connor, 2014; O’Connor, Sulloway, Robertson, & Kleindorfer, 2010). The adult fly has been present in the Galápagos since at least 1964 (Causton et al., 2006), but its larvae were first reported in Darwin's finch nests on Santa Cruz Island in 1997 (Fessl, Couri, & Tebbich, 2001) despite long‐term field study into Darwin's finches on other islands since 1973 (Grant & Grant, 2002). Field research found *P. downsi* requires *c*. 4–7 days to develop through three instar stages and reach pupation (Common, Dudaniec, Colombelli‐Négrel, & Kleindorfer, 2019; Kleindorfer, Peters, et al., 2014). In this newly evolving host–parasite system, mortality has been high in both *P. downsi* and its Darwin's finch hosts. On average, about 17% of *P. downsi* larvae die in the host nest and about 55 ± 6.2% of Darwin's finch nestlings die in the nest from *P. downsi* parasitism (Kleindorfer & Dudaniec, 2016). In addition to the high mortality it exerts, *P. downsi* parasitism has on average been killing nestling hosts at an earlier age of 5.4 ± 0.3 days post‐hatch in 2014 compared to 10.6 ± 0.5 days post‐hatch in 2004 (Kleindorfer, Peters, et al., 2014; O’Connor, Sulloway, et al., 2010). Questions remain as to how this earlier termination in parasite resources (nestling hosts) affects life cycle completion, body size and fecundity in *P. downsi*, and in turn, how the evolution of virulence may be affected.

In this study, we use 9 years of field data spanning a 12‐year period to examine changes in body size (an indirect measure of fecundity) in the dipteran ectoparasite, *P. downsi*, in response to the increasingly earlier death of its host. Given that there is a strong correlation between insect body size and fecundity (Armbruster & Hutchinson, 2002; Honěk, 1993; Preziosi et al., 1996; Tammaru, Esperk, & Castellanos, 2002), we analyse body size in adult *P. downsi* flies and pupae as indicators of *P. downsi* fecundity across years. If natural selection favours faster pupation and smaller body size as the consequence of earlier host mortality, we predict (a) smaller size in *P. downsi* pupae and adult flies from 2004 to 2016. If natural selection for smaller body size favours lower fecundity via trade‐offs between virulence and host resources, then we predict (b) a larger decrease in female body size relative to male body size in *P. downsi* adults. Together, this knowledge contributes to our understanding of how shifting host mortality in the natural environment directly selects for parasite body size as the consequence of faster pupation, which may lead to an indirect selection pressure on female fecundity.

## 2 MATERIALS AND METHODS

### 2.1 Study site and study species

We collected data from long‐term field study sites on the islands of Santa Cruz (Cimadom et al., 2014; Kleindorfer, 2007; Kleindorfer, Chapman, Winkler, & Sulloway, 2006) and Floreana (Kleindorfer, Peters, et al., 2014; O’Connor, Sulloway, et al., 2010) in the Galápagos Archipelago. We conducted field work during nine Darwin's finch breeding seasons spanning the months of February to April over 12 years: 2004, 2005, 2006, 2008, 2010, 2012, 2013, 2014 and 2016. On each island, study sites were located in both the arid lowland zone (El Garrapatero, −0.686479, −90.223775, and El Barranco, −0.739068, −90.301467 on Santa Cruz; habitat surrounding the town of Puerto Velasco Ibarra and La Loberia, −1.279932, −90.485927, on Floreana Island) and in highland *Scalesia* forest (Los Gemelos, −0.625982, −90.384829, on Santa Cruz; sites along the trail at the base of Cerro Pajas volcano, −1.299974, −90.452710, on Floreana Island). We sampled *P. downsi* from the following host species: small tree finch (*Camarhynchus parvulus*), hybrid *Camarhynchus* tree finch (cross between *C. pauper* and *C. parvulus* as well as introgressed individuals) (Kleindorfer, O’Connor, et al., 2014; Peters, Myers, Dudaniec, O'Connor, & Kleindorfer, 2017), medium tree finch (*C. pauper)*, woodpecker finch (*C. pallidus*), small ground finch (*Geospiza fuliginosa*) and medium ground finch (*G. fortis*) (Table S1). For analysis, we tested effects of host species and host genus (*Camarhynchus*, *Geospiza*) on *P. downsi* body size.

Adult *P. downsi* flies are vegetarian and feed on decaying plant material, so they do not pose a direct threat to Darwin's finches (Couri, 1985; Skidmore, 1985). However, the fly oviposits in active finch nests when the attending female is absent (Lahuatte et al., 2016; O’Connor, Robertson, & Kleindorfer, 2010; O'Connor, Robertson, & Kleindorfer, 2014), and multiple female flies may oviposit in a single nest (Dudaniec, Gardner, & Kleindorfer, 2010). After *P. downsi* eggs hatch, 1st‐instar larvae enter the nares and body cavities of the nestling and reside there to feed on blood and tissue (Fessl, Sinclair, & Kleindorfer, 2006). During the night, 2nd‐ and 3rd‐instar larvae emerge from the nest base to feed internally and externally on the body of nestlings (Fessl et al., 2006; Kleindorfer & Sulloway, 2016; O'Connor et al., 2014). After feeding for *c*. 4–7 days, 3rd‐instar larvae pupate in the nest base, forming a frothy cocoon, and adult flies emerge after 7–14 days (Kleindorfer, Peters, et al., 2014; Lahuatte et al., 2016). Although field research has found that *P. downsi* requires *c*. 4–7 days to develop through three instar stages and reach pupation (Kleindorfer, Peters, et al., 2014), laboratory studies have found that pupation occurs at *c*. 7–10 days (Bulgarella et al., 2017; Lahuatte et al., 2016). *Philornis downsi* parasitism causes higher than average nestling mortality in 10 out of 17 Darwin's finch species in which the interaction has been studied (Fessl et al., 2018; Kleindorfer & Dudaniec, 2016), with surviving nestlings commonly showing physical deformation of the naris into adulthood (Galligan & Kleindorfer, 2009; Heimpel, Hillstrom, Freund, Knutie, & Clayton, 2017; Kleindorfer, Custance, Peters Katharina, & Sulloway Frank, 2019; Kleindorfer & Dudaniec, 2016).

### 2.2 *Philornis downsi* collection from Darwin's finch nests

We monitored 116 Darwin's finch nests for nesting outcome using our well‐established field protocols (Kleindorfer, Peters, et al., 2014) in all sampling years except 2005. Upon nesting termination (fledging or death of the last nestling), each nest was collected in a sealed plastic bag, and all *P. downsi* larvae, pupae, empty puparia and adult flies were counted within 1–24 hr of collection. All *P. downsi* samples were stored in 90% ethanol immediately after counting. *Philornis downsi* intensity in the nest was measured as the total number of larvae, pupae, puparia and adult flies present upon collection of the nest. The sample size per year and host genus (*Camarhynchus, Geospiza*) is provided in Table S1.

### 2.3 *Philornis downsi* collection from McPhail traps

We placed a total of 114 McPhail Traps in the lowlands and highlands of Santa Cruz and Floreana Island to sample adult *P. downsi* flies in the years 2004, 2005, 2012, 2013 and 2014 (for details see Table S1). The McPhail traps were baited with a liquid lure of blended papaya, water and white sugar (following trapping protocol developed by P. Lincango and C. Causton) that was replaced every 7 days. Traps were hung in trees along 4 × 90 m transects, and flies were collected twice per week and stored in ethanol. In 2014 on Floreana Island, we placed 28 McPhail traps along four transects, seven traps per transect, at heights of 2–7 m. In other years and locations, traps were placed ad hoc every 50 m within 100 m × 200 m plots spanning a 2 km transect within study sites. We analysed data from 46 lowland traps and 68 highland traps (Table S1).

### 2.4 Pupa mass and size

Mass (g), length and width (mm) were measured for each pupa, as these measurements are known to be highly correlated with adult fly size (Gauld & Fitton, 1987; Quiroga & Reboreda, 2013; Shingleton, Mirth, & Bates, 2008; Stillwell, Dworkin, Shingleton, & Frankino, 2011), and can therefore be an indirect indicator of an individuals' fecundity upon maturity (Orozco & Bell, 1974; Preziosi & Fairbairn, 1996; Saino et al., 2017; Välimäki & Kaitala, 2007). Pupae cannot be sexed; therefore, these data could not be used for sexual dimorphism analysis but are useful when looking at general temporal shifts in body size in the *P. downsi* population. All pupae were removed from ethanol and placed on filter paper to dry for 30 s before taking measurements (Armbruster & Hutchinson, 2002). We measured the total mass of all intact pupae per nest and divided this by the number of pupae to calculate average pupa mass (Thomas, Fadul, Keller, & Chaudhury, 2018). The pupae were weighed to the nearest 0.001 g using an A&D HR‐200 Digital Analytical Balance. The length (mm) and width (mm) of the largest pupa per nest was measured using digital callipers. For analysis, we used the average mass per nest. Pupa mass was measured from the nests of 19 *C. parvulus* (268 pupae), 10 hybrid *Camarhynchus* tree finch (55 pupae), 25 *C. pauper* (332 pupae)*,* 57 *G. fuliginosa* (816 pupae) and 5 *G. fortis* (52 pupae) (Table S1).

### 2.5 Adult *P. downsi* size

We measured body size for 38 male and 38 female adult *P. downsi* from nests, and 34 male and 85 female adult *P. downsi* from McPhail traps. From the 43 nests and 114 McPhail traps sampled, we measured one male and one female adult fly unless there was only one sex present, in which case we used one sample per nest or trap. We visually sorted all fly specimens per sex for each nest or trap from smallest to largest and selected the median‐sized fly as the specimen for analysis. This approach was used because we measured the average pupa mass per nest and also to avoid any possible pseudoreplication due to genetic relatedness among the fly specimens. For each specimen, we used callipers with 0.1 mm accuracy to measure head length (mm), thorax length (mm) and abdomen length (mm), all measured with the specimen ventral side up; wing length (mm), measured from the base of the basicosta to the tip of the wing; and body length (mm), which was calculated from the values of head, thorax and abdomen length combined. For seven specimens, the head was missing due to previous DNA extractions; for 23 specimens, we only have data on body length as the specimens were destroyed for a separate study (Dudaniec et al., 2010). Therefore, sample size for head length (*N* = 188) and body length (*N* = 211) versus thorax, abdomen and wing length (*N* = 195) differ.

### 2.6 *Philornis downsi* body size and fecundity

To assess if the overall pattern of association between abdomen size/body size and number of eggs in *P. downsi* is comparable with the pattern reported in other Diptera studies, we collated published *r* and *r* ^2^ values across 17 studies (Table S2). Collated values were used to calculate average *r* ^2^ and 95% CI, and compared to the pattern found in *P. downsi.* We randomly sampled and dissected 10 female *P. downsi* specimens collected from McPhail traps at 4 m in the study area on Floreana Island in 2014 (Kleindorfer, Peters, Hohl, & Sulloway, 2016). One specimen was collected from a different trap and/or different collection week to ensure independence of data. Specimens were stored in 70% ethanol at room temperature for at least 24 hr before dissection and were dissected under a stereomicroscope at 16× magnification to count the total number of eggs present in ovaries (Malmqvist, Adler, & Strasevicius, 2004). We limit the sample size as the specimens are valuable intact for our long‐term study, and our aim is to test for an already established pattern of association in Diptera.

### 2.7 Statistical analysis

Data were analysed with SPSS version 25.0. The summary data are presented as mean ± standard error, unless otherwise stated. Data were checked for normality to satisfy requirements of parametric tests. We tested the association between abdomen size/body size and the number of eggs present in ovaries using linear regression analysis. We completed principal component analysis (PCA) on mean pupae mass, length and width to assess overall changes in pupae size. One principal component was retained, pupae size, which explained 85.96% of the total variation within these variables (Eigenvalue = 2.579) (Table S4). We used a generalized linear mixed model (GLMM) to test for an effect of year on pupae size with PC pupae size as the dependent variable, year, island and habitat as fixed factors, and species as a random factor. We then used linear regression to test for changes in pupae mass, length and width separately to investigate whether each variable displays a different pattern of change across time.

We explored adult fly size across years and in relation to sex (male, female). To assess overall changes in adult body size, we completed a PCA on abdomen length and body length. One principal component, fly size, was extracted which explained 91.4% of the variation within these three variables (Eigenvalue = 1.828) (Table S5). We used a GLMM to test for an effect of year on adult fly size using PC fly size as the dependent variable, and year, sex, year × sex as fixed factors and island as a random factor. There was no difference in body size of adult flies collected from Darwin's finch nests or McPhail traps (independent *t* test: head length: *t* = −0.003, *p* = .998; thorax length: *t* = −0.188, *p* = .851; abdomen length: *t* = 1.804, *p* = .074; wing length: *t* = 0.629, *p* = .530). Therefore, nest and trap data were pooled to test for the effect of year on *P. downsi* head, thorax, abdomen, wing and body length separately using linear regression analysis. We conducted linear regression analyses separated by sex to examine for sex differences. We derive all statistical conclusions from the GLMM analyses, but present individual regression analyses for comparative purposes.

## 3 RESULTS

### 3.1 Pupae size and mass

**Figure 1:**
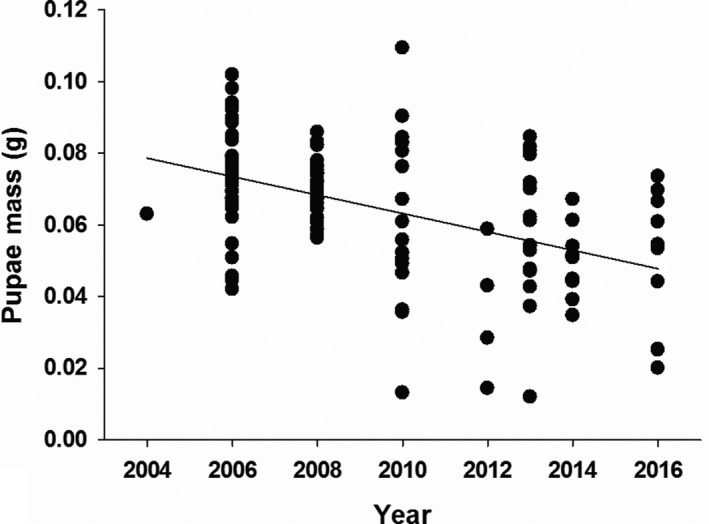
Mass (g) of *Philornis downsi* pupae collected from the nests of Darwin's finches between 2004 and 2016

Only the fixed factor year had a significant effect on pupae size (*F*_1,112_ = 30.814, *p* < .001); no other covariate or interaction term was related to *P. downsi* size (Table 1). There was no effect of species on pupae size (Wald *Z* = 0.540; *p* = .589). Using regression analysis, *P. downsi* pupae mass was negatively correlated with year (*F*_1,115_ = 30.709, *r* = −.461, *p* < .001, *N* = 115) (Figure 1), as was length (*F*_1,115_ = 12.086, *r* = −.310, *p* = .001, *N* = 115) and width (*F*_1,115_ = 33.450, *r* = −.476, *p* < .001, *N* = 115). Pupae mass decreased up to 32.9% (0.073 ± 0.003 g to 0.049 ± 0.006 g), pupae length by 5.8% (10.09 ± 0.12 to 9.50 ± 0.39 mm) and pupae width by 10.6% (4.17 ± 0.06 to 3.73 ± 0.15 mm). Since 2004, *P. downsi* pupae have become significantly lighter, shorter and narrower (Table S3). We found the same pattern when analysing the data separately for pupae collected from the nests of *Camarhynchus* finches (*N* = 53; mass: *F*_1,53_ = 17.476, *r* = −.502, *p* < .001; length: *F*_1,53_ = 10.476, *r* = −.409, *p* = .002; width: *F*_1,53_ = 28.045, *r* = −.592, *p* < .001) and *Geospiza* finches (*N* = 61; mass: *F*_1,61_ = 15.286, *r* = −.451, *p* < .001; length: *F*_1,61_ = 4.197, *r* = −.256, *p* = .045; width: *F*_1,61_ = 13.219, *r* = −.425, *p* = .001).

**Table 1:** Coefficients of the generalized linear mixed model of pupae size and adult fly size. The test statistic was *t* for fixed factors and *Z* for random factors

Response variable	Final model	Coefficients	Estimate	*SE*	Test statistic	*p*‐value
Pupae size						
PC pupae size	Year Island Habitat	Intercept	286.370	51.596	5.550	<.001
		Year	−0.143	0.026	−5.551	<.001
		Island	0.501	0.348	1.438	.153
		Habitat	0.103	0.227	0.453	.651
		Species	0.021	0.038	0.540	.589
Adult fly size						
PC adult size	Year Year × Sex Sex Island	Intercept	325.560	65.523	4.969	<.001
		Year	−0.162	0.033	−4.972	<.001
		Sex (female)^a^	−211.557	101.934	−2.075	.039
		Year × Sex	0.105	0.051	2.082	.039
		Island	0.090	0.142	−0.629	.530

a For Sex, male was set to zero.

### 3.2 Adult *P. downsi* size

We found a correlation between number of eggs and female body length (*F*_1,9_ = 7.085, *r* ^2^ = .47, *p* = .029, *N* = 10) (Figure S1) and abdomen length (*F*_1,9_ = 5.917, *r* ^2^ = .43, *p* = .041, *N* = 10) (Figure S2). We calculated the coefficient of determination (*r* ^2^) from 17 studies on Diptera (S2) that published the association between abdomen size and body size and the number of eggs. The overall *r* ^2^ in Diptera was .39 (95% CI 0.28–0.50), and hence, our values are in line with previous studies.

**Figure 2:**
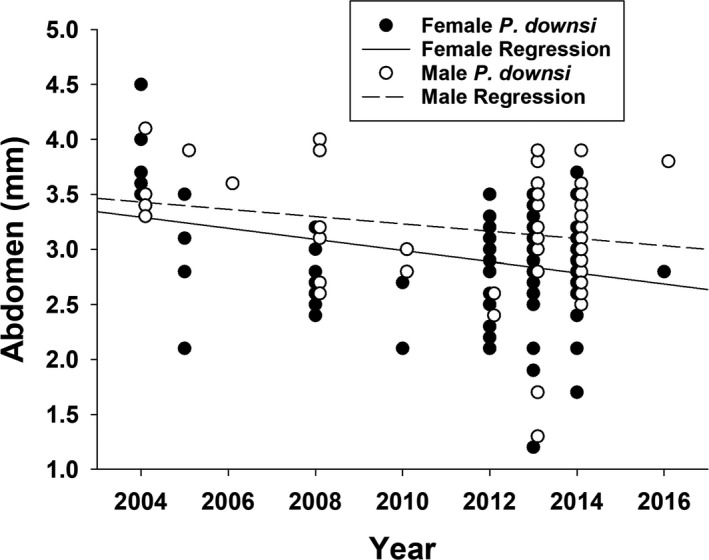
Abdomen length (mm) of male and female *Philornis downsi* adult flies collected from the nests of Darwin's finches and McPhail Traps between 2004 and 2016

We found an effect of year and sex on *P. downsi* adult size (Year: *F*_1,184_ = 19.435, *t* = −4.972, *p* < .001; Sex: *t* = −2.075, *p* = .039) (Table 1). Next, we explored each *P. downsi* body size variable. Combining adult males and females, there was a significant negative correlation between year and *P. downsi* head length (*F*_1,187_ = 8.394, *r* = −.208, *p* = .004, *N* = 188), thorax length (*F*_1,194_ = 12.438, *r* = −.246, *p* = .001, *N* = 195), abdomen length (*F*_1,194_ = 13.321, *r* = −.254, *p* < .001, *N* = 195) (Figure 2), wing length (*F*_1,194_ = 33.335, *r* = −.384, *p* < .001, *N* = 195) and total body length (*F*_1,187_ = 18.459, *r* = −.650, *p* < .001, *N* = 211). Adult flies were 7.6% smaller across the study period (8.44 ± 0.05 to 7.80 ± 0.17 mm), with abdomen length decreasing 7.2% (3.74 ± 0.11 to 3.47 ± 0.33 mm). We found similar patterns when analysing the data separately for *P. downsi* adults collected from McPhail traps (sexes pooled; *N* = 119; head: *F*_1,114_ = 4.356, *r* = −.193. *p* = .039; thorax: *F*_1,118_ = 3.423, *r* = −.169, *p* = .067; abdomen: *F*_1,118_ = 21.433, *r* = −.393, *p* < .001; wing: *F*_1,118_ = 6.236, *r* = −.225, *p* = .014; total body length: *F*_1,75_ = 23.140, *r* = −.412, *p* < .001) and *P. downsi* adults reared from pupae collected from Darwin's finch nests (sexes pooled; *N* = 72; head: *F*_1,72_ = 5.263, *r* = −.263. *p* = .025; thorax: *F*_1,75_ = 14.598, *r* = −.406, *p* < .001; abdomen: *F*_1,75_ = 3.397, *r* = −.210, *p* = .069; wing: *F*_1,75_ = 24.595, *r* = −.499, *p* < .001; total body length: *F*_1,72_ = 8.503, *r* = −.327, *p* = .005).

### 3.3 Male versus female adult fly size across years

Due to the significant interaction of year × sex on fly size (*F*_1,184_ = 4.336, *t* = 2.082, *p* = .039), we explored the differences in body size between the sexes in more detail. From 2004 to 2016, adult male *P. downsi* wing length and head length became smaller (wing: *F*_1,72_ = 19.147, *r* = −.463, *p* < .001, *N* = 72; head: *F*_1,69_ = 4.989, *r* = −.261, *p* = .029, *N* = 70) but there was no effect of year on male thorax length (*F*_1,69_ = 2.393, *r* = −.182, *p* = .126, *N* = 72) or abdomen length (*F*_1,71_ = 3.223, *r* = −.210, *p* = .077, *N* = 72) (Figure 2). Male head length decreased 15.0% across the study period (1.53 ± 0.10 to 1.30 ± 0.10 mm), and wing length decreased 7.0% (8.70 ± 0.25 to 8.09 ± 0.07 mm). Although not significant, there was a trend for smaller body length in adult males (*F*_1,69_ = 3.866, *r* = −.232, *p* = .53, *N* = 82). In adult female *P. downsi*, there was a negative correlation between year and wing length (*F*_1,121_ = 20.045, *r* = −.378, *p* < .001, *N* = 122), thorax length (*F*_1,121_ = 12.776, *r* = −.310, *p* = .001, *N* = 122), abdomen length (*F*_1,121_ = 12.591, *r* = −.308, *p* = .001, *N* = 122) (Figure 2) and body length (*F*_1,117_ = 20.058, *r* = −.384, *p* < .001, *N* = 129), but no effect of year on female head length (*F*_1,117_ = 3.533, *r* = .172, *p* = .063, *N* = 118). Across the study period, female thorax length decreased 18.0% (3.21 ± 0.18 to 2.63 ± 0.06 mm), abdomen length decreased by 25.6% (3.83 ± 0.14 to 2.85 ± 0.06 mm), and wing length decreased by 12.9% (8.59 ± 0.17 to 7.48 ± 0.09 mm).

## 4 DISCUSSION

Our findings show a change in *P. downsi* pupae and adult body size between 2004 and 2016 that is coincident with increasing in‐nest mortality in both parasite and host (Kleindorfer & Dudaniec, 2016) in a newly evolving host–parasite system. Across the time period sampled, we found up to a 25% reduction in *P. downsi* pupae and adult size but a greater size reduction in females than in males. Therefore, these results support evidence that natural selection favours faster pupation and smaller body size as a consequence of earlier host mortality in both sexes, and also that natural selection for smaller body size may favour lower fecundity because only abdomen size was smaller in females. Abdomen length in female insects is a trait functionally linked with fecundity. Female abdomen length decreased across years, whereas male abdomen length did not, which underscores fecundity changes in this system. Under conditions of early host death and high risk of in‐nest *P. downsi* mortality, natural selection favours larvae that pupate earlier and at a smaller body size. This smaller size at pupation results in adult flies with lower fecundity, supported by a correlation between female body size and the number of eggs. We do not know whether environmental plasticity or genetic changes explain variation in pupa and adult size, but both processes can be shaped by natural or sexual selection (Blanckenhorn, 2000; Perry, Schield, & Castoe, 2018). Smaller *P. downsi* body size and lower fecundity may have implications for parasite competition within host nests and the evolution of host virulence in Darwin's finches.

The impact of *P. downsi* on native and endemic Galápagos bird species cannot be overstated: nestlings are being heavily parasitized, nestling hosts experience intense competition within nests to avoid being parasitized, and most die in the nest (O’Connor, Robertson, et al., 2010; O'Connor et al., 2014). An average of 45% of parasitized birds fledge (Kleindorfer & Dudaniec, 2016) but those that survive often have bill abnormalities due to early instar larval feeding, which has implications for song characteristics and mate choice (Kleindorfer et al., 2019; Kleindorfer & Dudaniec, 2016; Kleindorfer & Sulloway, 2016). With the prediction that lower parasite fecundity should covary with lower virulence, Kleindorfer and Dudaniec (2016) found that the number of *P. downsi* in finch nests increased by 46% across the decade but that patterns of host mortality on both Floreana and Santa Cruz Island remained stable at a high *c*. 55% per year (Kleindorfer & Dudaniec, 2016; Kleindorfer, O’Connor, et al., 2014; Kleindorfer, Peters, et al., 2014). This suggests that forms of parasite resistance could be evolving in the host, or *P. downsi* is evolving to be less virulent—perhaps with the benefit of securing host resources for longer. Our data support the latter suggestion, with evidence for smaller *P. downsi* and lower *P. downsi* fecundity corresponding with earlier pupation in more recent years.

Given that *P. downsi* requires between 4 and 7 days to pupate in the field, the early death of host nestlings at *c*. 5 days post‐hatch is likely to exert strong selection pressures on larval development (Kleindorfer, Peters, et al., 2014; Lahuatte et al., 2016). Insect larvae are generally required to reach a critical mass in order to pupate, after which they can pupate immediately or continue to grow (Nijhout & Callier, 2015). Larvae can pupate faster and at a smaller size when starved after reaching that critical mass (Nijhout & Callier, 2015). Shorter development times have been linked with decreased body sizes in Dipterans (Butlin & Day, 1984; Lehmann et al., 2006), and larvae with resource termination or fewer resources during development were smaller as adults (Singh & Bala, 2009; Williams & Richardson, 1983). In *P. downsi*, earlier termination of host resources has likely led to shorter developmental periods, resulting in the smaller pupa size we observed. Understanding *P. downsi* developmental biology is critical for developing control strategies, with recent research gaining new insights into conditions that stimulate egg hatching in the field (Sage et al., 2018) and the effect of larval diet on pupal mass and developmental duration in a laboratory setting (Lahuatte et al., 2016). In the absence of a host, first‐instar *P. downsi* survived for up to 5 days, suggesting that larvae have the capacity to exploit and survive under conditions of unpredictable resources (Sage et al., 2018); however, body size and condition after starvation are not yet known.

Although decreasing *P. downsi* body size is coincident with early host termination, there may be other factors driving body size in this system. Density‐dependent parasite competition for limited resources may also affect developmental rate, body size and hence fecundity, a process that is well documented in Dipteran flies (Lieske & Zwick, 2008; Peckarsky & Cowan, 1991; Shiao & Yeh, 2008). In nests of Darwin's finches, *P. downsi* intensity varies considerably (Kleindorfer & Dudaniec, 2016), and the genetic relatedness of larvae indicates that multiple adult female flies oviposit eggs in a single nest (mean = 3.04 ± 0.21), and multiple males (mean = 1.97 ± 0.08) sire the offspring of each female, with an average of five offspring per female (range 1–24 offspring per female) (Dudaniec et al., 2010). Relatedness among larvae in finch nests is therefore very low, whereas studies have found that decreased genetic relatedness can increase competitive interactions within species, which in turn may compromise fitness (Frank, 1994). However, such interactions and any concurrent shifts in the genetic relatedness of *P. downsi* are yet to be examined.

Host switching by parasitizing more than one host life stage may increase development time due to suboptimal resources. Previously, *P. downsi* larvae were only present in Darwin's finch nests once the host nestlings had hatched (Fessl & Tebbich, 2002; O'Connor et al., 2014). However, in recent years, there have been a growing number of observations of *P. downsi* larvae in nests during the incubation phase suggesting that larvae are feeding on incubating females (Cimadom et al., 2016; Common et al., 2019). Incubating female finches have been found to express *P. downsi*‐specific antibodies (Huber et al., 2010), and females with higher antibody levels were found to have fewer parasites in their nest (Knutie et al., 2016; Koop, Owen, Knutie, Aguilar, & Clayton, 2013). Parasitizing incubating female finches may provide compromised nutrition for larvae due to the presence of *P. downsi*‐specific antibodies, protective feathers and behavioural adaptations such as the consumption and removal of larvae from nests (O’Connor, Robertson, et al., 2010). Despite these potential costs, earlier host infestation during female incubation may be an attempt to prolong larval developmental period due to the narrowing window of nestling resources imposed by earlier host mortality.

Male and female *P. downsi* showed different size trajectories as adults, with evidence for strong downward selection on abdomen size in females, but not in males. Findings from multiple studies have reported substantial benefits of being a larger‐bodied female (Blanckenhorn, 2000; Esperk, 2006), such as the associated increase in fecundity (Calder, 1996; Head, 1995; Honěk, 1993; Tammaru et al., 2002). Notably, the significant decrease in female abdomen length we observed suggests an impact of earlier pupation on fly fecundity. Natural selection for faster pupation can have fecundity impacts when body size and specific body regions linked with reproductive output are affected by development time (Olsson et al., 2002; Pincheira‐Donoso & Hunt, 2017; Wickman & Karlsson, 1989; Winkler et al., 2012). Downward fecundity selection has been documented far less frequently than upward fecundity selection (Nunney, 1996; Preziosi & Fairbairn, 1996; Quintero‐Fong et al., 2018; Reeve & Fairbairn, 1999), and most evidence for downward selection comes from laboratory studies rather than natural systems (Preziosi & Fairbairn, 1996). It is important to note limitations with using the magnitude of female‐biased sexual size dimorphism to determine the strength of fecundity selection as discussed by Pincheira‐Donoso and Hunt (2017). Due to the effects of sexual selection on sexual size dimorphism (Cox & Calsbeek, 2009), research into the strength of sexual selection in *P. downsi* populations should be conducted to determine the driving factors for changing male and female body size.

Host–parasite co‐evolution is rarely observed in natural systems, and biological invasions by parasites offer an opportunity to explore co‐evolutionary processes (Feis, Goedknegt, Thieltges, Buschbaum, & Wegner, 2016). Understanding the effects of downward fecundity selection on female oviposition behaviour, larval competition within nests and virulence patterns in Darwin's finches will further unravel the host–parasite co‐evolutionary dynamics occurring in this system. This study provides further understanding of host–parasite co‐evolution during invasion and parasite trade‐offs of fecundity and nutrition under strong natural selection.

## CONFLICT OF INTEREST

The authors declare no conflict of interest.
