# Supplementary material for: Evidence for rapid downward fecundity selection in an ectoparasite (Philornis downsi) with earlier host mortality in Darwin’s finches
Source: J Evol Biol. 2020 Feb 7;33(4):524–33. doi: 10.1111/jeb.13588 (PMC7217188; doi:10.1111/jeb.13588)
Supplement: Supplementary file 2 [file JEB-33-524-s002.docx]

**Supplementary Material**

**Table S1**

Summary data for all samples used in analysis.

**Table S2**

Collated published coefficients of determination of the association between body size and fecundity.

| **Reference** | **r^2^** | **r** |
| --- | --- | --- |
| Honěk (1993) | 0.856 |  |
| McCann et al. (2009) | 0.05, 0.73 |  |
| Armbruster and Hutchinson (2002) | 0.61, 0.83 |  |
| Blackmore and Lord (2000) | 0.071, 0.12 |  |
| Frankino and Juliano (1999) | 0.636 |  |
| Blay and Yuval (1999) | 0.25, 0.6 |  |
| Malmqvist et al. (2004) | 0.312, 0.783 |  |
| Briegel (1990) |  | 0.60 |
| Sivinski (1993) |  | 0.86 |
| Steinwascher (1984) |  | 0.34, 0.64 |
| Xue and Ali (1994) |  | 0.39 |
| Reigada and Godoy (2005) |  | 0.30 |
| Kolluru and Zuk (2001) |  | 0.32, 0.36 |
| Riback and Godoy (2008b), Riback and Godoy (2008a) |  | 0.55, 0.56 |
| Gião and Godoy (2006) |  | 0.63, 0.73 |

**Table S3**

*Philornis downsi* pupae size separated by genus. The mean mass and size of *P. downsi* pupae per year sampled from Darwin’s finch nests, whereby the genus of the host species was either *Camarhynchus* or *Geospiza*. The data are shown for *P. downsi* pupa mass (g), length (mm) and width (mm). Data are shown as mean ± SE calculated from the average per nest.

|  | *P. downsi* collected from  *Camarhynchus* nests | | | *P. downsi* collected from  *Geospiza* nests | | |
| --- | --- | --- | --- | --- | --- | --- |
|  | Mass | Width | Length | Mass | Width | Length |
| 2006 | 0.07 ± 0.00 | 4.27 ± 0.08 | 10.21 ± 0.21 | 0.07 ± 0.00 | 4.11 ± 0.09 | 10.02 ± 0.15 |
| 2008 | 0.07± 0.00 | 3.91 ± 0.07 | 9.69 ± 0.16 | 0.07 ± 0.00 | 3.98 ± 0.07 | 10.21 ± 0.16 |
| 2010 | 0.06 ± 0.01 | 3.54 ± 0.18 | 9.14 ± 0.55 | 0.07 ± 0.01 | 3.81 ± 0.09 | 9.74 ± 0.27 |
| 2012 | 0.03 ± 0.01 | 2.90 ± 0.32 | 7.70 ± 0.51 | - | - | - |
| 2013 | 0.06 ± 0.01 | 3.58 ± 0.12 | 9.24 ± 0.31 | 0.07 ± 0.01 | 3.74 ± 0.12 | 9.84 ± 0.15 |
| 2014 | 0.05 ± 0.00 | 3.38 ± 0.17 | 8.88 ± 0.37 | 0.05 ± 0.01 | 3.63 ± 0.22 | 9.23 ± 0.27 |
| 2016 | - | - | - | 0.05 ± 0.01 | 3.73 ± 0.15 | 9.50 ± 0.39 |

**Table S4**

Component coefficients and communalities of variables *Philornis downsi* pupae mean mass (g), length (mm) and width (mm) loaded with extracted Principal Component Pupae Size. Major loadings for each item are in bold.

|  | Component coefficients | Communalities |
| --- | --- | --- |
| Mean mass | **0.932** | 0.869 |
| Length | **0.931** | 0.867 |
| Width | **0.918** | 0.843 |

**Table S5**

Component coefficients and communalities of variables *Philornis downsi* adult abdomen length (mm) and body length (mm) loaded with extracted Principal Component Adult Fly Size. Major loadings for each item are in bold.

|  | Component coefficients | Communalities |
| --- | --- | --- |
| Abdomen length | **0.956** | 0.914 |
| Body length | **0.956** | 0.914 |


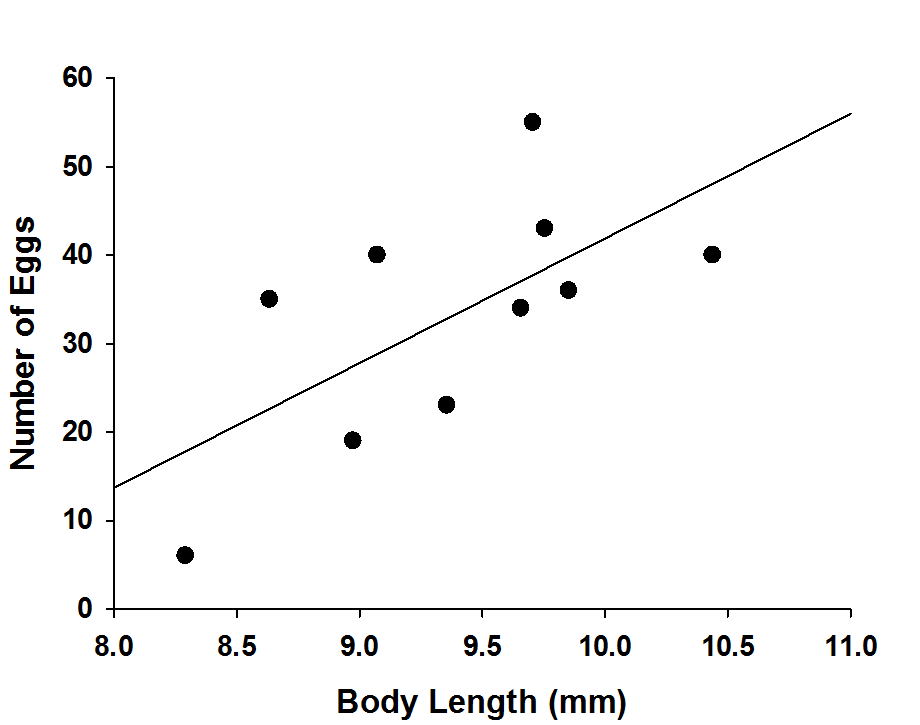


**Figure S1**

Correlation between body length (mm) and number of eggs of female *Philornis downsi* adult flies collected from 4m McPhail Traps on Floreana Island in 2014.

**
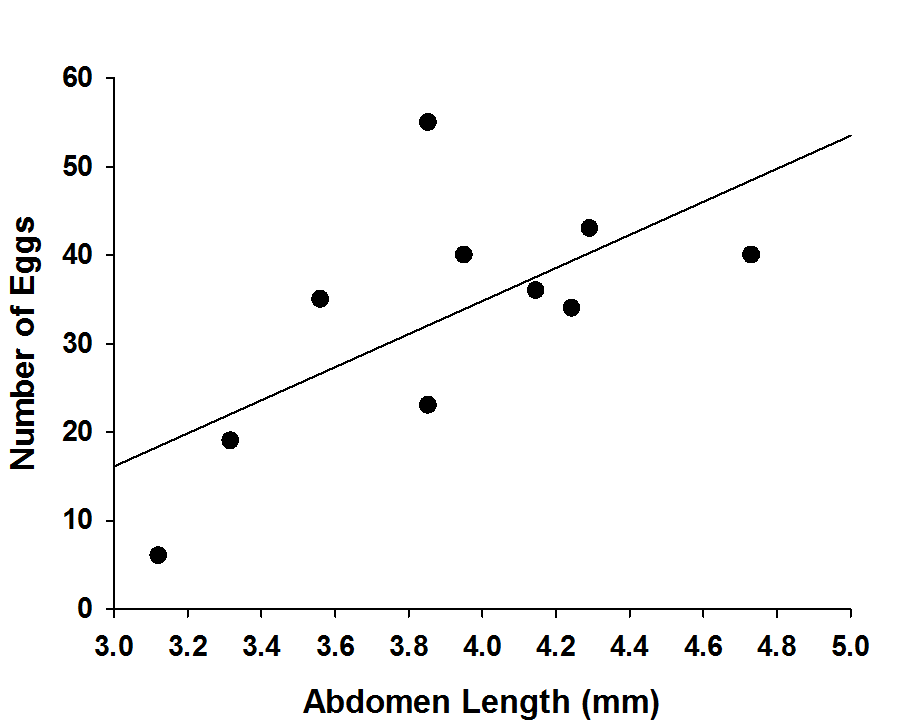
**

**Figure S2**

Correlation between abdomen length (mm) and number of eggs of female *Philornis downsi* adult flies collected from 4m McPhail traps on Floreana Island in 2014.
